# Clinical and immunological features of laryngeal cryptococcosis

**DOI:** 10.1590/S1678-9946202466040

**Published:** 2024-07-08

**Authors:** Vítor Falcão de Oliveira, Mariane Taborda, Mateus Bach Santa Catarina, Wdson Luis Lima Kruschewsky, Marjorie Marini Rapozo, Thais Queiroz da Rocha, Carla Pagliari, Adriana Satie Gonçalves Kono Magri, Marcello Mihailenko Chaves Magri, Miriam Nacagami Soto

**Affiliations:** 1 Universidade de São Paulo, Faculdade de Medicina, Hospital das Clínicas, Departamento de Moléstias Infecciosas e Parasitárias, São Paulo, São Paulo, Brazil; 2 Universidade de São Paulo, Faculdade de Medicina, Hospital das Clínicas Departamento de Patologia, São Paulo, São Paulo, Brazil

**Keywords:** Laryngeal cryptococcosis, Cryptococcosis, Immunopathology

## Abstract

The literature holds few descriptions on immune response findings for laryngeal cryptococcosis. Immunology has been more extensively described in cases involving the central nervous system and the lungs, although many of these studies were conducted in animal models. We aimed to analyze the clinical and immunological characteristics of three patients with laryngeal cryptococcosis. We observed a weak participation of the innate immune response, whereas adaptive immunity showed the predominance of a Th2-type response over a Th1-type response. Most cases occur in male older adults with immunosuppressive conditions, of which HIV infection was absent. Hoarseness configured the main symptom. We found a disease that was restricted to the larynx and possibly the lungs by contiguity. Patients with hoarseness and lesions in nasal endoscopy should be investigated for cryptococcosis by a biopsy of the larynx, including with negative serum cryptococcal antigen. The immunological aspects of our findings of laryngeal involvement resembled those in the most commonly affected systems.

## INTRODUCTION

Cryptococcal infections are prevalent worldwide^1^. *Cryptococcus* sp., including *Cryptococcus neoformans* and *Cryptococcus gattii*, are ubiquitous and are the main causes of cryptococcosis in humans^2^. *C. neoformans* is more commonly found in immunocompromised patients, whereas *C. gattii* is more frequently associated with immunocompetent hosts^3^.

*Cryptococcus* sp. have multiple immune system evasion mechanisms, and dissemination of the fungus may occur virtually to all organs, but the central nervous system (CNS) and the lungs are the main affected systems^4^. It is widely recognized that the host’s primary defense mechanism for solving cryptococcosis involves cell-mediated immunity, which suppresses the growth of yeasts in the lungs^5^. While T helper (Th)1 cells are crucial in initiating a protective immune response against cryptococcal infection, Th2 cells produce interleukin IL-4, IL-13, and IL-5. These cytokines have a detrimental effect on immune responses to cryptococcosis infections^6^. Nonetheless, some studies in humans have found no correlation between Th2 immune responses and disease amelioration^7,8^.

Laryngeal cryptococcosis is rare, with few reported cases^9-13^. Its main symptom involves persistent or progressive hoarseness and lesions that predominantly occur on the true vocal cords^9^. The literature holds few descriptions about immune response findings to laryngeal cryptococcosis. Immunology is more extensively described in cases involving the CNS and the lungs, although many of these studies were conducted in animal models. Due to the uncommon involvement of the larynx, immune responses are likely to differ, especially considering that the main risk factor in case series involves immunocompetent patients using inhaled corticosteroids^9-12,14,15^. We aimed to analyze the clinical and immunological characteristics of three patients with laryngeal cryptococcosis.

## CASE REPORTS

### Case 1

**Figure 1:**
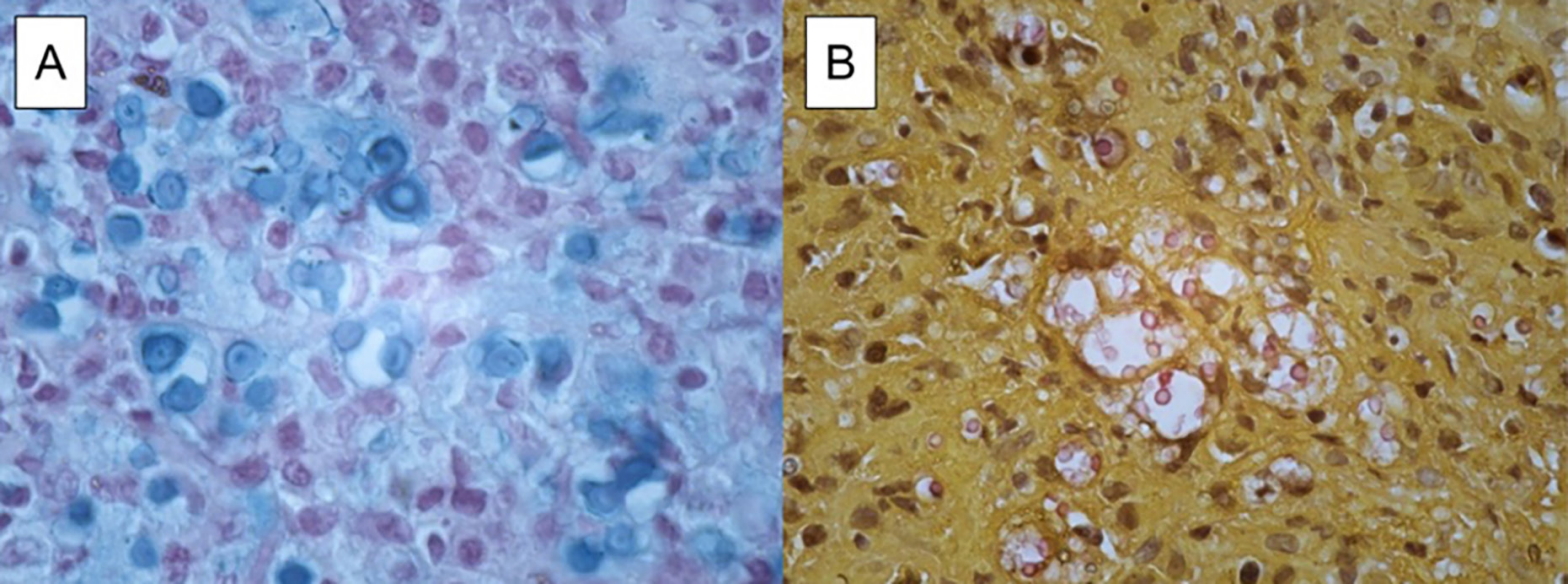
A) Alcian Blue staining shows the acidic mucin in the *Cryptococcus* spp. capsule (Case 1); B) Mucicarmine stain is positive for *Cryptococcus* spp. (Case 2).

A 67-year-old Brazilian man underwent a liver transplant in March 2022 due to cryptogenic biliary cirrhosis. He has a history of hypertension, diabetes mellitus, and smoking. His immunosuppressive drugs include prednisone, tacrolimus, and mycophenolate mofetil. He was admitted to the infectious disease ward in December 2022 with a six-month history of sudden and progressive hoarseness, without associated symptoms and with a normal physical examination. Bronchoscopy showed an irregular vegetative-infiltrative lesion affecting the middle third of his left vocal cord and spanning the ipsilateral ventricular band and posterior commissure, without significant limiting airflow. Histological analysis showed a chronic granulomatous infiltrate with multiple giant cells, and Grocott and Alcian Blue stains were positive for *Cryptococcus* spp. (Figure 1A).

This patient showed unremarkable laboratory findings, including negative HIV serology. Fungal serology for *Histoplasma, Paracoccidioides*, and serum *Cryptococcus* Antigen (CrAg) Latex Agglutination Test (LA) were negative. Chest computed tomography (CT) showed ground-glass opacities, foci of consolidation, and centrilobular micronodules in the posterior segment of the right upper and lower lobes, more pronounced on the right. Additionally, he had a well-delimited non-calcified pulmonary nodule in the lingular superior segment measuring 0.9 cm. Brain CT was normal. The examination of cerebrospinal fluid (CSF) was performed, and showed normal results. Direct mycological examination and fungal culture were negative. The serum and CSF *Cryptococcus* antigen tests were also negative.

**Figure 2:**
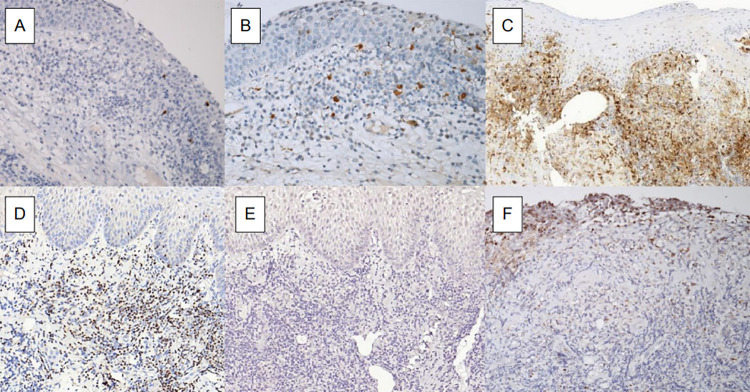
Immunohistochemical expression of: A) CD1a; B) S100 protein; C) CD4; D) GATA-3; E) IFN-γ; and F) IL-17.

The analysis of the innate immune system showed immunohistochemical reactions for CD68 with sparse macrophages in areas of lymphocyte predominance, and the reaction for CD56 was very scarce, indicating low dendritic cell density. The adaptive immunity was evinced by immunohistochemical reactions for CD3, CD4, and CD8, showing a predominance of CD4 positive T-cells and the transcription factor GATA3 (Figure 2D), a marker of Th2-activated lymphocytes. Markers TNF-alpha and IFN-gamma obtained negative results (Figure 2E), interpreted as a lack of a Th1 inflammatory response (Table 1).

**Table 1:** Clinical characteristics and immunology of patients with laryngeal cryptococcosis.

	Case 1	Case 2	Case 3
**Characteristic**			
Age (years)	67	69	81
Sex	Male	Male	Male
Main comorbidities	Liver transplant	Diabetes mellitus	Pulmonary Langerhans cell histiocytosis.
Inhaled corticosteroids	No	No	Yes
HIV infection	No	No	No
Clinical findings	Hoarseness	Hoarseness, dysphagia, shortness of breath, productive cough, and weight loss	Hoarseness, cough, weight loss
Type of lesion	Vegetative	Granulomatous	Granulomatous
Serum cryptococcal antigen	Negative	Negative	Negative
Treatment	Liposomal amphotericin-B, followed by fluconazole	Liposomal amphotericin-B, followed by fluconazole	Fluconazole
Outcomes	Clinical improvement	Clinical stability	Clinical improvement
**Immunology**			
CD1a	Negative	Very scarce	Very scarce
S100 protein	Negative	Negative	Scarce
CD68	Abundant	Abundant	Abundant
CD56	Very scarce	Very scarce	Very scarce
IL-17	Negative	Scarce	Scarce
Tbet	Negative	Negative	Mild
TNF-α	Scarce	Mild	Mild
IFN-γ	Negative	Scarce	Negative
GATA3	Mild	Mild	Mild

The patient was treated with Liposomal Amphotericin-B (L-AmB) at a dose of 3 mg/kg/day for one week, followed by 450 mg of fluconazole daily. He was discharged after two weeks of hospitalization. After six months of treatment, the patient reported complete improvement of hoarseness. The patient is awaiting a new bronchoscopy to conclude the treatment.

### Case 2

A 69-year-old Brazilian man worked as a farmer and had hypertension, diabetes, and benign prostatic hyperplasia. He was a former smoker and had a history of alcoholism. His last occupation was as a lumberman. He was admitted to the infectious diseases ward with a seven-month history of progressive hoarseness, dysphagia, shortness of breath, productive cough, and weight loss.

Bronchoscopy showed a granulomatous lesion on his right vocal fold with almost complete airway obstruction. The patient underwent urgent tracheostomy, and the lesion was biopsied. Histological analysis showed encapsulated yeast-like structures on mucicarmine stain, compatible with *Cryptococcus* spp. (Figure 1B).

This patient showed unremarkable laboratory findings, including negative HIV serology. Fungal serology for *Histoplasma, Paracoccidioides*, and serum CrAg LA were also negative. Chest CT showed ground-glass opacities, foci of lung consolidation, and grossly nodular lesions in the lower lobes and lingula.

We observed a negative S100 protein-positive cell and very few CD1a and CD56, which reflects the weak participation of his innate immune response. The expression of IL-17 was negative in the lymphoid tissue. The adaptive immunity showed the predominance of CD4 positive T-cells (Figure 2C) and of transcription factor GATA3. TNF-α and IFN-γ markers showed mild and scarce levels, respectively, which were interpreted as lack of a Th1 inflammatory response (Table 1).

The patient was treated with L-AmB at a dose of 3 mg/kg/day for two weeks, followed by fluconazole 600 mg/day for two months. At the last appointment, the patient showed partial improvement of the lesion after an initial six-month of antifungal treatment.

### Case 3

An 81-year-old Brazilian man with pulmonary Langerhans cell histiocytosis was admitted to the infectious disease ward with a two -month history of progressive hoarseness and cough. He used inhaled corticosteroids.

Nasal endoscopy showed a granulomatous lesion in the anterior commissure and middle thirds of the vocal folds, interarytenoid area, and ventricular bands. Histological analysis showed encapsulated yeast-like structures on mucicarmine and Grocott stains. This finding suggests *Cryptococcus* spp.

This patient showed unremarkable laboratory findings, including negative HIV serology. Fungal serology for *Histoplasma, Paracoccidioides*, and serum CrAg LA were also negative. Chest CT showed nodular opacities in the middle and upper lobes associated with conglomeration of pulmonary fibrosis, mediastinal and hilar lymph node enlargement, and bronchiectasis.

We observed a weak participation of the innate immune response with CD1a and S100 protein (Figures 2A and 2B). Adaptive immunity showed a predominance of a Th2-type responses when compared with Th1-type responses due to the predominant expression of the transcription factor GATA3 (Table 1). The lymphoid tissue scarcely expressed IL-17 (Figure 2F).

The patient was treated with fluconazole 600 mg/day for two months. He showed slight clinical improvement. Nasal endoscopic examination showed partial improvement in the lesion on the right vocal fold. The treatment schedule will continue for six months.

## DISCUSSION

This study performed an immunopathological characterization of laryngeal cryptococcosis. We observed that a weak participation of the innate immune response and the predominance of a Th2-type response in adaptive immunity are significant to understand the immunological aspects of the disease. The main symptom of hoarseness is also important for clinical diagnosis. Additionally, our finding of a disease primarily restricted to the larynx (with a possible extension to the lungs by contiguity) suggests the localized nature of the infection and its potential progression.

In laryngeal cryptococcosis, a minority of patients was immunocompromised, and the main immunosuppression condition was HIV infection^9^. Although all patients in our study had some degree of immunosuppression, none lived with an HIV infection.

Regarding immunocompetent hosts, inhaled corticosteroid use configures a risk factor^16^. This can explain why the cryptococcal infection is more localized in the larynx. No patient had a disseminated disease. All cases probably included pulmonary findings by contiguity, however bronchoalveolar lavage was not performed. Worrall*et al*. showed that no patients had CNS involvement^9^.

In a way, this localized involvement may explain the low sensitivity of the serum CrAg (sensitivity = 39%) in diagnosing cryptococcosis^9^. All patients in our study had a negative antigen test. Diagnosis was made by histopathology in all cases, with visualization of cryptococcal yeasts.

The primary virulence factors of *Cryptococcus neoformans*, including urease, laccase, and capsules, play a pivotal role in promoting the aggregation of immature dendritic cells and inducing nonprotective Th2 immune reactions. By adaptive immunity, CD4+ T cells facilitate the transcription and expression of relevant cytokines and chemokines, coordinating fungal clearance and protecting naive mice. Tregs, a subset of CD4+ T cells, critically mediate peripheral tolerance and modulate immune responses. Tregs play both positive and negative roles in fungal infections. They disrupt the dynamic balance of Th1/Th2/Th17 cells, reduce the expression of Th2 cytokines, and inhibit the differentiation of Th17 cells. Treg-deficient mice show an increase in the production of immunoglobulin E, eosinophils, and Th2 cytokines such as IL-4, IL-5, and IL-13. The expression of the transcription factor GATA3 is up- or downregulated during Th2 or Th1 cell differentiation, respectively. Furthermore, GATA3 induces Th2 differentiation and represses Th1 differentiation. Beyond CD4+ T cells, CD8+ T cells also exert a significant role in host immunity against *C. neoformans* infections by producing tumor necrosis factor gamma (TNF-γ), which inhibits and eliminates pathogens by direct contact^17^. Regarding immune responses, in the absence of macrophages and dendritic cells responsible for innate immunity, polymorphonuclear leukocytes and B cells accumulate in the tissue but are unable to control the fungal infection. Their increasing presence is associated with excess damage to the host^18,19^. Furthermore, the increased expression of Th1 cytokines, such as TNF-α and IFN-γ, enhances fungal control. However, *C. neoformans* can active shift the Th1-Th2 balance toward a Th2 profile, and the predominant Th2 cells can promote fungal growth and dissemination^8,17,18^. Our study showed that laryngeal cryptococcosis was also immunologically Th2-dominant.

The immunopathology of cryptococcosis in CNS and pulmonary disorders has been more extensively studied. Regarding immunology, our findings of laryngeal involvement resembled to those observed in other common sites. Nonetheless, our case series is quite limited and all cases showed possible pulmonary involvement due to contiguity. Further studies are necessary to enhance the immunological characterization of laryngeal cryptococcosis.

## CONCLUSION

In conclusion, our study described the immunopathological features of laryngeal cryptococcosis, which included a weak participation of the innate immune response and a predominance of a Th2-type response. Laryngeal involvement demonstrated a localized disease with diagnosis primarily based on histology. We recommend that patients presenting hoarseness and lesions during nasal endoscopy should be investigated for cryptococcosis through biopsy of the larynx, even if serum cryptococcal antigen testing yields negative results.
